# Nursing now and always: evidence for the implementation of the Nursing Now campaign*

**DOI:** 10.1590/1518-8345.4553.3388

**Published:** 2020-11-06

**Authors:** Isabel Amélia Costa Mendes, Carla Aparecida Arena Ventura, Manoel Carlos Neri da Silva, Valeria Lerch Lunardi, Ítalo Rodolfo Silva, Sara Soares dos Santos

**Affiliations:** 1 Universidade de São Paulo, Escola de Enfermagem de Ribeirão Preto, PAHO/WHO Collaborating Centre for Nursing Research Development, Ribeirão Preto, SP, Brazil.; 2 Scholarship holder at the Conselho Nacional de Desenvolvimento Científico e Tecnológico (CNPq), Brazil.; 3 Conselho Federal de Enfermagem, Brasília, DF, Brazil.; 4 Universidade Federal do Rio Grande, Departamento de Enfermagem, Rio Grande, RS, Brazil.; 5 Universidade Federal do Rio de Janeiro, Campus Macaé, Macaé, RJ, Brazil.

**Keywords:** Nursing, Global Health, Health Workforce, Leadership, Staff Development, Education, Nursing, Enfermagem, Saúde Global, Pessoal de Saúde, Liderança, Desenvolvimento de Pessoal, Educação em Enfermagem, Enfermería, Salud Global, Personal de Salud, Liderazgo, Desarrollo de Personal, Educación em Enfermería

## Abstract

**Objective:**

to identify the guiding axes of the documents that grounded the Nursing Now campaign and relate the recommendations of these documents to the campaign goals.

**Method:**

documentary research, based on the analysis of the documents that promoted the Nursing Now campaign. The data were collected between March and April 2020, using a form structured into: background, scope, challenges and potentials of health/nursing professionals and recommendations for the future.

**Results:**

the challenges and the need for investments in the health and nursing workforce to achieve the Sustainable Development Goals were evidenced. The report of the High Level Commission on health Employment and Economic Growth presents important recommendations, also introduced in the Triple Impact Report and in the Strategic Directions for Nursing and Midwifery, converging to the goals of the Nursing Now campaign, stimulating a profile of nurses with technical, political and leadership skills, engaged in health policy-making, and the effectiveness of their practice is clear to the entire society.

**Conclusion:**

knowledge about the dynamics of the factors that converged to the development of the *Nursing Now* campaign may be a condition for achieving its goals. This reality reveals evidence that global health will not be ensured without strengthening Nursing first.

## Abstract (pt)

**Objetivo:**

identificar os eixos norteadores dos documentos que deram origem à campanha *Nursing Now* e relacionar as recomendações desses documentos aos objetivos da campanha.

**Método:**

pesquisa documental, realizada a partir de análise dos documentos que fomentaram a campanha *Nursing Now*. Os dados foram coletados entre março e abril de 2020, mediante formulário estruturado em *background*, escopo, desafios e potencialidades dos profissionais da saúde/enfermagem e recomendações para o futuro.

**Resultados:**

foram evidenciados os desafios e a imprescindibilidade de investimentos na força de trabalho em saúde e Enfermagem para o atingimento dos Objetivos de Desenvolvimento Sustentável. O Relatório da *High Level Commission on Health Employment and Economic Growth* apresenta recomendações importantes, também introduzidas no *Triple Impact Report* e nas *Strategic Directions for Nursing and Midwifery,* confluindo para os objetivos da Campanha *Nursing Now*, que estimulam um perfil de enfermeiro com competências técnicas, políticas, de liderança, envolvido na construção de políticas de saúde, cuja efetividade da prática seja evidenciada para toda a sociedade.

**Conclusão:**

conhecimento sobre a dinâmica dos fatores que convergiram para o desenvolvimento da campanha *Nursing Now* pode ser condição para alcançar seus objetivos. Depreendem-se, dessa realidade, evidências de que a saúde global não será garantida sem, antes, fortalecer a Enfermagem.

## Abstract (es)

**Objetivo:**

identificar los ejes orientadores de los documentos que dieron origen a la campaña *Nursing Now* y relacionar las recomendaciones emanadas de esos documentos con los objetivos de la campaña.

**Método:**

investigación documental, desarrollada a partir del análisis de los documentos que fomentaron la campaña *Nursing Now*. Los datos fueron recogidos entre marzo y abril de 2020, mediante formulario estructurado en: *background*, alcance, retos y potencialidades de los profesionales sanitarios/enfermeros y recomendaciones para el futuro.

**Resultados:**

fueron evidenciados los desafíos y la importancia fundamental de inversiones en la fuerza de trabajo en salud y Enfermería para el logro de los Objetivos de Desarrollo Sostenible. El Informe de la *High Level Commission on Health Employment and Economic Growth* presenta recomendaciones importantes, también introducidas en el *Triple Impact Report* y en las *Strategic Directions for Nursing and Midwifery in the Americas,* confluyendo a los objetivos de la campaña *Nursing Now*, estimulando un perfil de enfermero con competencias técnicas, políticas, de liderazgo, involucrado en la construcción de políticas de salud, cuya efectividad de su práctica sea evidenciada para toda la sociedad.

**Conclusión:**

conocer la dinámica de los factores que convergieron hacia el desarrollo de la campaña *Nursing Now* puede ser una condición para alcanzar sus objetivos. Se deduce de esta realidad evidencias de que la salud global no será garantizada sin haber fortalecido la Enfermería.

## Introduction

In 2020, Nursing, with approximately 27.9 million professionals, represents 59% of all health professionals worldwide, constituting the majority group in the health area^(^ 1 ^)^. Contradictorily, despite this numerical relevance, the estimated global shortage of nurses is about nine million by 2030^(^ 2 ^)^.

It should be highlighted that, for the sake of standardization, the term *nurses* is used in this article to designate all members of the nursing team, in accordance with the documents of the World Health Organization (WHO) analyzed in this study. The use of the term *Nursing*, in turn, designates both the profession and the academic discipline.

As a historical milestone, it is important, initially, to highlight WHO’s 72^nd^ World Health Assembly, which recognized 2020 as the International Year of Nurses and Midwives, not only to honor the 200^th^ anniversary of the birth of Florence Nightingale, but to reaffirm and grant visibility to the daily contribution of Nursing to the health and well-being of people^(^ 3 ^)^. Therefore, WHO underlines that nurses are fundamental to achieving the goal of *leaving no one behind*, in a global context of seeking to implement the Sustainable Development Goals (SDG) by 2030. Among the strategies to achieve the SDG, universal health coverage and access, so that everyone is entitled to health services^(^ 2 ^-^ 4 ^)^, requires not only the strengthening and qualification of nursing education, as investments for the continuation of these workers in professional practice.

Thus, the vital contribution of Nursing to the achievement of global and Brazilian goals related to the priorities on the global health agenda is recognized, including universal access, mental health and non-communicable diseases, emergency preparedness and response and patient safety, always focusing on people-centered care^(^ 1 ^,^ 5 ^)^.

Despite its representativeness and the acknowledged relevance of its work, however, Nursing is still invisible and undervalued in many aspects, especially in health policy-making. Hence, in addition to the recognition and valuation of its numerical importance, it is essential to value high-quality Nursing for leadership, policy and decision-making, participation in decision-making processes, and action ranging from planning to care provided to specific population groups, including the fight against pandemics^(^ 5 ^-^ 8 ^)^, as occurs in the context of COVID-19.

In this perspective, it is imperative to invest in the qualification of nurses to prepare them to cope with health problems worldwide, so that their contributions and potentials are properly understood. Among the manifestations of the recognition of Nursing as a key profession for health and for the achievement of universal health coverage and access, on February 27, 2018, the global *Nursing Now* campaign was launched, implemented in collaboration between the International Council of nurses (ICN) and WHO, with the support of the Burdett Trust for Nursing, a coalition of nurses and other health advocates^(^ 9 ^)^.

The global campaign was based on evidence from key documents that demonstrate the relevance of Nursing to global health: Report of the High-Level Commission on Health Employment and Economic Growth-working for Health and Growth^(^ 10 ^)^, Triple Impact Report^(^ 11 ^)^ and Global Strategic Directions for Nursing and Midwifery (2016-2020)^(^ 12 ^)^.

In this context, it is worth noting that the goals of the *Nursing Now* campaign, to be met between 2018 and 2020, are: 1) Increase investments in the improvement of teaching, professional development, standards, regulations, and the terms and employment conditions of nurses; (2) Increase and improve the dissemination of effective and innovative approaches in the field of nursing; 3) Intensify the influence of nurses and midwives in global and Brazilian health policies, as part of broader efforts to ensure that human resources for health are more engaged in decision-making processes; (4) Enhance leadership positions occupied by nurses, and multiply opportunities for development at all levels; and (5) Provide an expanded evidence base for the decision-makers and policy-makers about where Nursing can have the greatest impact, what prevents nurses from reaching their full potential, and how to deal with these obstacles.

Therefore, the *Nursing Now* campaign sets relevant and challenging objectives, emphasizing the fundamental importance of Nursing leadership for the achievement of local, Brazilian and global health targets. It is based on the assumption that health agendas will not be successful without nurses in leadership positions, in the different spaces for health policy-making and decision-making, and who perform their functions with greater effectiveness. Thus, at least 75% of countries should have a Chief Nursing Officer as part of their most qualified teams in health management and policies. Therefore, greater investments in the training of nurses with political and “policy” skills is fundamental^(^ 7 ^)^.

In this context, the *Nursing Now* campaign also seeks to demonstrate the individual and collective value of Nursing, supporting research that documents the visibility and socioeconomic impact of Nursing on the healthcare quality and costs. It aims to reset the boundaries of practice, valuing the philosophical assumptions of the nursing discipline and the social mission of the profession^(^ 13 ^)^. Thus, the campaign has been implemented in 123 countries, with decentralized goals, but which align with the global objectives of the campaign in each nation involved^(^ 9 ^)^.

This being a unique moment for global Nursing and in view of the importance of understanding the creation and implementation context of the *Nursing Now* campaign, the objective in this article is to identify the guiding axes of the documents that grounded the Nursing Now campaign and relate the recommendations in those documents with the campaign goals.

## Method

Descriptive and documentary research^(^ 14 ^)^, based on the analysis of documents that encouraged the creation and implementation of the *Nursing Now* campaign. The identification of the documents was based on the reading of the seminal document that officialized the launch of the global campaign.

In this process, it was essential to recover historical materials that referred to the political and health scenario, at the time of the preparation of the documents that motivated the planning and development of the *Nursing Now* campaign, aiming at an in-depth understanding of its contributions and importance for the creation and implementation of the campaign.

Therefore, three official reports that promoted the *Nursing Now* campaign constituted the scope of analysis, namely: Report of the High-Level Commission on health Employment and Economic Growth-working for Health and Growth^(^ 10 ^)^; Triple Impact: How developing nursing will improve health, promote gender equality and support economic growth^(^ 11 ^)^; Strategic Directions for Nursing and Midwifery - 2016-2020^(^ 12 ^)^.

These historical documents were accessed from the official websites of the UN, WHO and ICN. Together, these documents establish the campaign, justifying the need for a specific movement to value Nursing as a means of effective investment in strengthening health systems and improving the health conditions of the population, around the globe.

Three independent researchers repeatedly read these reports and individually completed a data collection script they had prepared in advance. In case of disagreement between the analyses, the material could be analyzed by a fourth researcher. That was not necessary though. The script consisted of the topics: background, scope, challenges and potential of health/nursing professionals’ work, as well as recommendations for the future.

**Figure 1:**
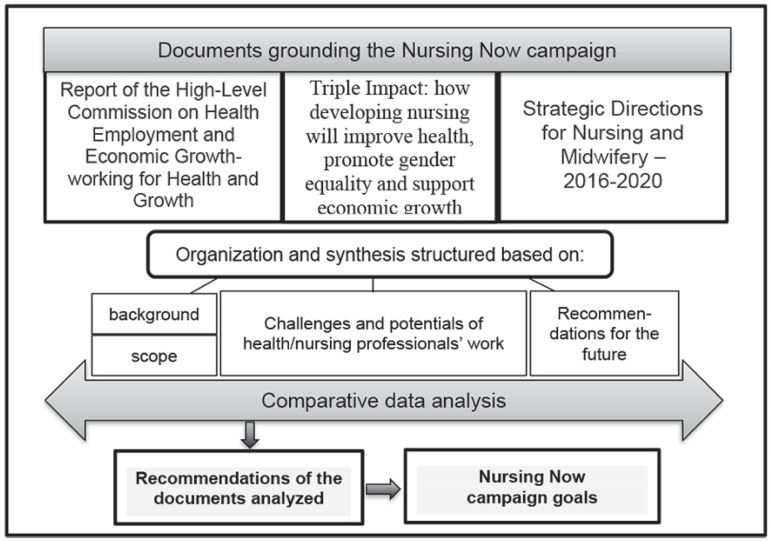
Data organization and analysis.

The data collection script made it easier to synthesize in order to describe the data and identify the guiding axes that promoted the *Nursing Now* campaign. After completing each topic in the script, for each analyzed report, the comparative analysis of the data started. Thus, for example, a synthesis of the topic “recommendations for the future” could be elaborated among the documents (reports). In the next step, the recommendations of the reports that structured the campaign (guiding axes) were identified, relating them to each of the *Nursing Now* campaign goals. Figure 1 illustrates the organization and analysis of the research.

The research data were collected between March and April 2020. Being official documents in the public domain, collected through online access, registration or approval of the research by an Ethics Committee for Research involving human beings was deemed unnecessary.

## Results

The information collected in the analysis process, based on the documents^(^ 10 ^-^ 12 ^)^ that grounded and motivated the Nursing Now campaign, have been detailed below.

In Figure 2, the 2016 report of the UN High Level Commission on Health Employment and Economic Growth sets an important background for the global political agenda, focusing on the valuation of the health workforce. It presents relevant interrelationships between investments in the health sector and the achievement of universal health coverage and different SDG.

**Figure 2:** Background, scope, potential and challenges for the work of health professionals and recommendations of the Report of the High-Level Commission on Health Employment and Economic Growth – working for health and growth

Report of the High-Level Commission on Health Employment and Economic Growth-working for Health and Growth
**Background**
Commission established by United Nations Secretary General Ban Ki-moon in March 2016. Its task was to make recommendations to stimulate and guide the creation of at least 40 million new jobs in the health and social sectors and to reduce the lack of 18 million workers, especially in low and middle-income countries, by 2030. The results of this work reflect the partnership with the International Labour Organization (ILO), Organization for Economic Cooperation and Development (OECD) and WHO.
**Scope of health professionals’ work**
The Commission qualified the launch of its report as a unique opportunity to advance in the political commitment to achieve the SDG*, particularly SDG 1 (poverty elimination), 3 (health and welfare), 4 (quality education), 5 (gender equality) and 8 (decent work and economic growth), through investments in the local and global health workforce.
**Potential of health professionals’ work**
Acknowledgement of the health sector as a key economic sector and employment generator. Between 2000 and 2014, employment in health and social work grew by 48%, while jobs in the industry and agriculture dropped. The demand for health services is expected to grow, creating millions of new jobs. Economic development depends on healthy populations. Around a quarter of growth in low and middle-income countries between 2000 and 2011 resulted from improvements in health; the estimated return on investments in health is 9 to 1; one extra year in life expectancy raises GDP per capita by about 4%. In countries with a high fertility rate, bringing down the child mortality rate can positively influence family planning decisions, contributing to a faster demographic transition, associated with economic benefits called demographic dividends. Investments in health systems have multiplier effects that strengthen inclusive economic growth, also through decent jobs. Strategic investments in health systems are fundamental, including in the health workforce and in the promotion of economic growth, via cohesion and social protection, innovation and health security.
**Challenges for health professionals’ work**
The population is growing and the world faces a decrease in the number of health workers. The lack of skilled workers constrains job creation in the sector. Investments in health education/training are needed to promote more inclusive economic growth.
**Recommendations**
1. *Job creation* – stimulate investments in creating decent jobs in the health sector, especially for women and young people, with the necessary skills, in the right numbers and places. The Commission calls for urgent actions to develop job market policies to foster the demand for a sustainable health workforce. Government policies need to address systemic issues that result in recurring losses in the health job market. 2. *Gender equality rights* – maximize women’s economic participation, institutionalizing their leadership and addressing gender biases and inequalities in education and work. Women are the main care providers, including in humanitarian crises and conflicts. Hence, the health sector is an increasing employer for women and can contribute to gender equality. Gender inequalities, physical and sexual violence and harassment remain important challenges for health workers. 3. *Education, training and skills* – value high-quality, transformative education and continuing learning so that health workers have skills to face people’s health needs and work to their full potential. In that sense, countries can prioritize investments in education, focusing on building locally relevant competencies. Addressing geographical inequities is a priority, and demographic transitions present opportunities to strengthen youth education for health jobs. 4. *Health service delivery and organization* – Reform health service models concentrated on hospitals and focus on prevention and efficient delivery of high-quality, integrated, community-based and people-centered primary care, paying special attention to vulnerable areas. 5. *Technology* – explore the power of Information and Communication Technologies (ICT) that strengthen health education, people-centered health services and health information systems. The rapid technological changes are changing the nature of health services and new health professionals emerge with skills to handle the ICT. Digital technologies also provide opportunities to improve people’s access to health services and health systems’ response to the needs of individuals and communities. 6. *Humanitarian crises* – ensure investments in key international health regulations, including the skills development of health workers in humanitarian crisis and public health emergency situations, aiming to ensure the protection and security of health workers in different settings. Countries should build the capacity of their health workforce and health systems to detect and respond to public health risks and emergencies. In conflict settings, public health crises exacerbate the difficulties for offering basic care. 7. *Financing and fiscal resources* – raise adequate national and international funding from public and private sources and consider the funding of health reforms through investments in appropriate skills and decent working conditions. Societal dialogue and political commitment are crucial to drive appropriate macroeconomic reforms and health funding policies. 8. *Partnership and cooperation* – promote intersectoral collaboration at the national, regional and international levels, through the engagement of civil society, unions and other organizations of health workers and the private sector. Align international cooperation to support investments in the health workforce. 9. *International migration* – advance the international recognition of health professionals’ qualifications to optimize their skills use, increasing the benefits from and reducing the negative effects of health workers’ migration, safeguarding migrants’ rights. 10. *Reliable evidence* – undertake robust research on the health markets, using reliable methodologies to strengthen the evidence, reliability of the data and resulting actions.

* SDG = Sustainable development goals

Figure 3 displays key points of the Triple Impact Report, highlighting the challenges the nursing workforce faces, and primarily the unique nature of its contribution to the achievement of SDG 3, 5 and 8. Understanding this document’s guiding premises is fundamental to understand the motivations that resulted in the Nursing Now campaign.

**Figure 3:** Background, scope, potential and challenges for the work of health professionals and recommendations of Triple Impact: how developing nursing will improve health, promote gender equality and support economic growth

Triple Impact: how developing nursing will improve health, promote gender equality and support economic growth
**Background**
Report organized by the All-Party Parliamentary Group on Global Health in the United Kingdom. Its starting point was the goal countries signed in 2015, assuming the commitment to guarantee universal health access/coverage, adopting the view that “nobody should be left behind”. This report argues that universal health coverage cannot be achieved without strengthening Nursing; it highlights the need to increase the number, as well as the understanding that its contribution is understood, so as to enable nurses to work at their full potential. In that perspective, the report argues that the strengthening of Nursing will have a triple impact: improving health, promoting gender equality and supporting economic growth.
**Scope of nursing work**
Nurses play different roles, in varying circumstances and contexts, through a unique combination of people-centered competencies and humanitarian values; they provide and manage care, work with families and communities and play a central role in public health, disease and infection control. In diverse contexts, nurses are the primary or sole professionals people see in health care. Part of the community, they share its culture and are alert to social, individual and programmatic vulnerabilities.
**Potential of nursing work**
Nurses are the largest part of the professional health workforce. Achieving the goal of universal health coverage/access globally depends on these professionals’ comprehensive action. There is important innovation and creativity in nursing care, with great potential not yet used that will ensure that citizens have better access to health care. The increase in the number of nurses and the use of Nursing’s full potential will result in a triple impact of improving health, gender equality and economic growth.
**Challenges for nursing work**
In many contexts, Nursing remains undervalued and its contribution underestimated. These professionals still face problems in the team, poor infrastructure and inappropriate education and training. All of these factors can result in care of inferior quality; in this context, they are frequently unable to fully practice their skills and share their knowledge. They also get few opportunities to develop their leadership, hold leading functions and influence policy making.
**Recommendations**
1. Strengthen Nursing and make it central to health policies. A high-level global summit on Nursing should be convened, consisting of political and health leaders outside Nursing, to raise awareness of the opportunities and potential of Nursing, create political commitment and a establish a process for supporting development. This should be part of a long-term initiative that embraces all other recommendations. 2. Support plans to increase the number of nurses being trained and employed globally. The WHO Global Strategy on Human Resources for Health, Workforce 2030, adopted by member states in 2016, proposes a framework for making the most effective use of health workers, as well as investment plans to address their shortage. A) Develop plans in partnership with low and middle-income countries to support their health workforce; B) Reaffirm support for the WHO Global Code of Practice on the International Recruitment of Health Personnel, offering support for the training and employment of health professionals in their countries of origin; C) Assess the impact of health professionals’ leaving the European Union on health and the health system and take mitigation actions. 3. Develop nurse leaders and Nursing leadership. They are needed in the right places to ensure that the distinctive Nursing perspective is included in health policy-making and decision-making. A) Establish a global program to develop nurse leaders who are truly engaged in policy-making and decision-making. B) Ensure that countries have nurse leadership posts throughout their structures and organizations. 4. Enable nurses to work to their full potential. Cultural, regulatory and legislative barriers need to be identified and removed so that good practices are shared and applied. 5. Collect and disseminate evidence of the impact of Nursing on health access, quality and costs, aiming to ensure that it is incorporated in policies. It is important to undertake new assessments to demonstrate the impact of Nursing at scale. A) Develop research to join evidence and start new research on how and when the improvement of Nursing care contributes to universal health access. B) Ensure that existing and future research results are widely disseminated and understood in order to influence practice and policies. 6. Develop Nursing to have a triple impact on health, gender equality and economies. Investment in Nursing – the vast majority of whom are women – will help empower them economically and as community leaders, strengthening local economies. A) Adapt development policies to implement programs that simultaneously address SDG 3, 5 and 8, focusing on health, gender equality and inclusive and sustainable economic growth. 7. Promote partnerships and mutual learning between the United Kingdom and other countries. A) Expand the DFID Health Partnership Scheme, including more nurses.

In Figure 4, the relevant elements of the document Strategic Directions for Nursing and Midwifery - 2016-2020 are presented, which display the continuity of Nursing’s response to different World Health Assembly resolutions aiming for the strengthening of Nursing and Midwifery.

**Figure 4:** Background, scope, potential and challenges for the work of health professionals and recommendations in the document Strategic Directions for Nursing and Midwifery – 2016-2020.

Strategic Directions for Nursing and Midwifery – 2016-2020
**Background**
In 2000, the 54^th^ World Health Assembly, by resolution WHA54.12 on Strengthening Nursing and Midwifery, requested the Director-General to prepare a plan of action for strengthening Nursing and Midwifery. As a result, the first Strategic Directions for Nursing and Midwifery were published in 2002 and updated in 2011. Since then, other resolutions on strengthening Nursing and Midwifery services have been passed by the World Health Assembly. The strategic directions provide decision makers, nurses and other stakeholders at every care level with a comprehensive reference framework for collaborative action to strengthen the capacity for Nursing and Midwifery development. In that context, WHO continued to act on that commitment: in May 2014, the 67th World Health Assembly adopted resolution WHA 67.24 on the Follow-up of the Recife Political Declaration on Human Resources for Health: renewed commitments towards universal health coverage. In paragraph 4(2) of that resolution, the member states requested the Director-General of WHO to present a new global strategy for human resources for health – The Global Strategy on Human Resources for Health Workforce 2030, which provided the foundation for the elaboration of these Strategic Directions. The Strategic Directions were developed through an extensive consultative process, including experts from all WHO regions, academicians, educational teaching institutions, government Chief Nursing Officers, policy-makers, WHO Collaborating Centers for Nursing and Midwifery Development, students, NGOs and professional associations. The document builds on the following guiding principles: ethical action – planning and offering care based on equity, integrity, fairness and respectful practice in the context of human rights; relevance – development of education programs, research, services and systems guided by health needs, evidence and strategic priorities; ownership – adoption of a flexible and strategic approach that ensures effective leadership, management and capacity building, as well as transparency, engagement and involvement mechanisms of all beneficiaries; partnership – joint work on common objectives; quality – adoption of mechanisms and standards based on evidence and best practices, through education and research.
**Scope of nursing work**
The global disease burden is increasing and gaining complexity, including emerging and re-emerging noncommunicable diseases. Nursing is critical in the delivery of essential health services and thus strengthens the health systems. Acting as individuals, members and coordinators of interprofessional teams, nurses bring people-centered care closer to the communities where it is needed most, improving the health outcomes and service effectiveness. They are active in promoting and maintaining the health and wellbeing of the elderly population. At the same time, they contribute to reductions in newborn, infant and maternal mortality. They are responsible for a wide range of hospital services, ranging from accident and emergency to palliative care. They are core in crisis and post-crisis situations, contributing to risk communication, response planning and multisectoral participation aspects of different programs, providing services ranging from trauma management to mental health.
**Potential of nursing work**
Nurses and midwives constitute more than 50% of the health workforce. In that scenario, there is demonstrable evidence supporting the contribution of Nursing to the strengthening of health systems through increased patient satisfaction, decreased morbidity and mortality rates, stabilization of financial systems through the reduction of readmissions and length of stay, among other conditions such as hospital-acquired infections, providing an overall contribution to patient wellbeing and safety. The utilization of the nursing workforce is cost-benefit. Nurses are the first responders to complex humanitarian crises and disasters, protecting and advocating for the community; and serve as team coordinators. Nursing interventions in the treatment of chronic conditions stimulate improved treatment adherence. Studies also show that family planning and maternal and child health interventions can avert a total of 83% of maternal and neonatal deaths.
**Challenges for nursing work**
There is continued need to strengthen the quality of nursing education, in response to unhealthy lifestyles, risk factor reduction and provision of different disease and infection-related interventions. Nursing education and practice takes place in an environment of constant technological changes, and its promotion is an important element for the future. Technological advancement can support transformational outcomes of more integrated, high-quality and knowledge and evidence-based approaches. In response to the challenges nursing and midwifery face, the document emphasizes that robust leadership, governance and accountability are essential. Strategic planning based on collecting and monitoring data and country indicators can contribute to effective education and recruitment, as well as to the retention and effective management of the nursing workforce.
**Recommendations**
1. Ensuring an educated, competent and motivated Nursing workforce within effective and responsive health systems at all levels and in all service settings. Objectives: to educate, recruit and retain a sufficient number of nurses with appropriate competencies, equipped with the necessary resources and governed by professional regulation. Strategies: to align investments and coordinate plans for the development of nursing in workforce coordination; in continuing education; regulation and in guaranteeing healthy practice environments. 2. Optimizing policy development, effective leadership, management and governance. Objectives: to favor the active participation of nursing leaders at every level of policy formulation, program planning development and implementation, including evidence generation for informed decision-making. Strategies: to prepare Nursing leaders to meet the challenges of health systems, ensuring their competence in all aspects of Nursing development, including policy development and evidence generation, in order to improve the quality of education and Nursing service provision. 3. Working together to maximize the capacities and potentials of Nursing through interprofessional collaborative partnerships and continuing professional development. Objectives: to optimize the impact of Nursing on health systems at all levels through intra- and interprofessional collaboration. Strategies: to delineate, monitor and evaluate roles, functions and responsibilities of the Nursing workforce to advance collaborative education and practice. 4. Mobilizing political initiatives to invest in effective scientific evidence on Nursing workforce development. Objectives: to establish structures that enable the empowerment of nurses in order to achieve effective engagement and contribute to health policy development, aiming to increase the quantity and quality of nursing workforce services. Strategies: to build political support and the highest levels of health systems and with civil society to ensure that the policies created are in line with the achievement of the universal health coverage and Sustainable Development Goals.

In Figure 5, the recommendations of the documents that were analyzed are directly related with the Nursing Now campaign goals.

**Figure 5:** Distribution of the Nursing Now campaign goals and their relationship with the recommendations of the documents Report of the High-Level Commission on health Employment and Economic Growth-working for Health and Growth; Triple Impact: How developing nursing will improve health, promote gender equality and supports economic growth; Strategic Directions for Nursing and Midwifery –2016-2020

Nursing Now campaign goals	Recommendations of the documents analyzed
1. Expand investments in improving the teaching, professional development, standards, regulations and employment conditions of nurses.	Recommendation 1 High Level Commission Report. Job creation; Recommendation 2 High Level Commission Report. Gender equality rights; Recommendation 3 High Level Commission Report. Education, training and skills; Recommendation 4 High Level Commission Report. Health service delivery and organization; Recommendation 5 High Level Commission Report. Technology; Recommendation 6 High Level Commission Report. Humanitarian crises; Recommendation 7 High Level Commission Report. Funding and fiscal resources. Recommendation 8 High Level Commission Report. Partnerships and cooperation. Recommendation 9 High Level Commission Report. International Migration. Recommendation 2 Triple Impact Report. Support plans to increase the number of trained and employed nurses globally. Recommendation 4 Triple Impact Report. Enable nurses to work to their full potential. Thematic Area 1 Strategic Directions. Ensure an educated, competent and motivated nursing workforce in effective and responsive health systems at all levels and services.
2. Increase and improve the dissemination of effective and innovative practices in nursing.	Recommendation 10 High Level Commission Report. Reliable evidence. Recommendation 5 Triple Impact Report. Collect and disseminate evidence on the impact of nursing on access, quality and costs, in order to ensure its incorporation into policies. Recommendation 6 Triple Impact Report. Develop nursing to have a triple impact on health, gender equality and economies. Thematic Area 4 Strategic Directions. Mobilize political initiatives for investments in effective scientific evidence on the development of the nursing workforce.
3. Intensify the influence of nurses and midwives on the global and national health policy as part of more broad-ranging efforts to ensure that human resources for health are more engaged in decision-making processes.	Recommendation 1 Triple Impact Report. Raise the profile of nursing, making it central to health policies. Thematic Area 3 Strategic Directions. Work together to maximize nursing skills and potentials through interprofessional partnerships and collaborations and continuous professional development. Thematic Area 4 Strategic Directions. Mobilize political initiatives for investments in effective scientific evidence on the development of the nursing workforce.
4. Enhance leadership positions occupied by nurses and multiply development opportunities at all levels.	Recommendation 1 Triple Impact Report. Raise the profile of nursing, making it central to health policies. Recommendation 3 Triple Impact Report. Develop nursing leaders and leadership in nursing. Thematic Area 2 Strategic Directions. Optimize policy-making, effective leadership, management and governance.
5. Provide an expanded evidence base for decision makers and policy makers on how and when nursing can have the greatest impact and what prevents nurses from reaching their potential to manage these obstacles.	Recommendation 2 High Level Commission Report. Gender equality rights - maximize women’s economic participation by institutionalizing their leadership and dealing with gender biases and inequalities in education and the job market. Recommendation 10 High Level Commission Report. Reliable evidence - develop robust research and analyses on health markets, using reliable metrics and methodologies to strengthen evidence, reliability of data, and resulting actions. Recommendation 5 Triple Impact Report. Collect and disseminate evidence on the impact of nursing on access, quality and costs, in order to ensure its incorporation into policies. Thematic Area 4 Strategic Directions. Mobilize political initiatives for investments in effective scientific evidence on the development of the nursing workforce.

Therefore, the data support that each goal of the Nursing Now campaign is based on complementary evidence in the seminal documents analyzed for the overall strengthening of nursing.

## Discussion

The three documents analyzed in this article emphasize the challenge imposed on the international community to invest in the health and Nursing workforce as a condition for achieving different SDG^(^ 4 ^)^ and, specifically, targets for the advancement of global health^(^ 10 ^-^ 12 ^)^. Thus, the Report of the High Level Commission on Health Employment and Economic Growth^(^ 10 ^)^ presents a global diagnosis of the health workforce conditions. With the participation of an ICN representative in the Commission, this document presents important recommendations that were also introduced in the Triple Impact Report^(^ 11 ^)^ and in the Strategic Directions for Nursing and Midwifery^(^ 12 ^)^.

It should be taken into account that the objectives of the Nursing Now campaign and the recommendations evidenced in the documents studied, are interconnected, which also mobilizes and enables their analysis and discussion in an interrelated way. The fundamental issue of achieving universal health access and coverage by 2030, in view of the implementation of the SDG, demonstrates and implies the need to make visible and disseminate the relevance of Nursing, demonstrating its socioeconomic value^(^ 7 ^)^, understanding Nursing not as a cost to institutions, but as an investment^(^ 13 ^,^ 15 ^)^. This necessary demonstration implies, however, facing the challenge of opposing arguments that support the thesis of this investment, knowing beforehand that two priorities of health managers are always at the top of the list: cost reduction without changing the quality of care and improvement of patient outcomes while maintaining reasonable costs^(^ 16 ^)^. In this negotiation, the assertiveness of nurse leaders’ arguments needs to be based on the best evidence on the effectiveness of Nursing work, through evidence deriving from comparative analyses between similar conditions of performance, with and without nurses, proving the added value of this workforce in the health indicators of the patients under their care.

In addition, the defense will be convincing if the nurse leader demonstrates to the health manager the value of Nursing in achieving health outcomes in economic terms. In the same perspective of budget containment, nurses who ensure the patient’s safe return of the patient successfully develop the alternative of a shorter hospital stay^(^ 17 ^-^ 19 ^)^.

In this perspective, it should be emphasized that nurses need to be attentive to capitalize in favor of Nursing on all these initiatives and successful interventions, ensuring that these records, in economic value, are computed in those organizations as cost savings resulting from nursing work, without loss of quality and ensuring care continuity at the primary level, preventing readmissions and ensuring the return of the health system user to society and work.

The first goal of the Nursing Now campaign recommends investments in the improvement of teaching, professional development, standards, regulations and employment conditions of nurses. It is directly related to Recommendations 1, 2, 3, 4, 5, 6, 7, 8 and 9 of the Report of the High Level Commission on Health Employment and Economic Growth as well as Recommendation 4 of the Triple Impact Report and Thematic Area 1 of the Strategic Directions for Nursing and Midwifery. Therefore, most recommendations of the High Level Commission on Health Employment and Economic Growth were related to this campaign goal. In this sense, for the sake of investments in professional development and employment conditions, it is essential to invest in job creation, in the provision and organization of health services. In the meantime, it is paramount to invest in the Nursing workforce, fundamentally in high-quality, modern, dynamic and relevant teaching, which is attractive to future nurses, avoiding, as early as in the education process, possible drop-out movements^(^ 11 ^)^.

Thus, the goal is teaching based on evidence of the best and most modern practices for learning^(^ 1 ^)^, with prepared and sufficient teachers who acknowledge Nursing education as a challenge, with relevant *curricula* and sufficient clinical and management experiences and training. An education that guarantees Brazilian and international recognition of its competence and quality, ensuring rights in possible immigration processes, with preparation focused on the health needs of the populations, the most vulnerable groups and areas, centered on people, but also with skills to act in situations of humanitarian crises, particularly considering the workers’ rights and requirements of protection and security. It is important for this education to present the students, future nurses, with how they should act as leaders, providing role models, broadening horizons and opportunities, as well as the prestige of Nursing, in such a way that future generations will be able to advocate for improvements in services, policies, and opportunities for constant training as a pre-condition for self-confidence, quality improvements, security, and stimulus to action, as well as the creation and implementation of positive changes in the outcomes of the population’s health and well-being.

In addition, plans should be supported to increase the number of trained and employed nurses around the world, ensuring the appropriate training and motivation of this workforce for work at all health levels and services. Among the recommendations, the unique importance of Nursing work in situations of humanitarian crises and the challenge that is imposed on countries in cases of international migration of nurses are highlighted. Still within the scope of this first goals, for the advancement of nursing education, it is relevant to value quality and continued education throughout working life, exploring the power of information and communication technologies, based on the recognition of gender equality rights and aiming to maximize women’s economic participation in the job market.

In this sense and strongly articulated to the necessary investment in high-quality education, motivating and mobilizing future nurses, health systems need to be responsive, with the necessary perspective of attractive jobs, not only to welcome young newly qualified nursing workers, but to ensure their permanence and motivation for the practice of the profession. Therefore, jobs and openings need to created that are recognized as decent, with salaries that permit fulfilling nurses’ basic needs, whose work organization is also decent, fair, appropriate, and compatible in terms of sufficiency, quality and value of human resources, and materials and equipment essential for their performance^(^ 1 ^)^. Despite different possible problems, Nursing practice at the regional, Brazilian and international level may share the competencies of its workers, requiring continuing development, which implies the recognition and valuation of nursing with lifelong experience and the strengthening of clinical leaders, highlighting that good leaders create other leaders^(^ 1 ^)^. Achieving this objective will only be possible when enhancing funding and fiscal resources, as well as through local, regional and international intersectoral collaboration and partnerships.

Campaign goal 2 aims to *increase and improve the dissemination of effective and innovative practices in nursing* and is related to Recommendation 10 of the Report of the High Level Commission on Health Employment and Economic Growth, with Recommendations 5 and 6 of the Triple Impact Report and with Thematic Area 4 of the Strategic Directions. In this perspective, the documents highlight the relevance of research development with robust evidence that underpins the dissemination of effective and innovative Nursing practices. Therefore, research on the impact of Nursing on health service access, quality and costs is suggested, as well as to increase the gender equality and improve the countries’ economic conditions. Expanded efforts are needed to disseminate the evidence in formats that better reach the service professionals, as well as the policy makers.

The traditional mode of responsibility the researcher complies with by merely publishing results in journals is outdated, given its insufficiency due to lack of capillarity and the characteristics of the postmodern society in the digital age: direct methods need to be explored to increase the quality and speed of scientific dissemination and translation of knowledge.

The third goal advocates nurses and midwives’ greater influence in global and Brazilian health policies, as part of broader efforts to ensure that human health resources are involved in decision-making processes and is related to Recommendation 1 of the Triple Impact Report and thematic areas 3 and 4 of the Strategic Directions for Nursing and Midwifery. The recommendations reinforce the need to strengthen the profile of Nursing, making it central to health policies^(^ 20 ^)^. They also focus on the importance of Nursing and its work through interprofessional collaborations, as well as investment in search of scientific evidence that strengthens the development of the Nursing workforce. For this, “breaking silences” is needed^(^ 6 ^)^, rupturing walls that sometimes Nursing itself has built in a siege process, conquering other health professionals, politicians, legislators and health leaders as allies for the work of nurses, so that they are allowed to freely use their full potential and competence, not only in a technical, but essentially in a political perspective.

Goal 4 reinforces the need for more nurses in leadership positions and more development opportunities at all levels and relates to Recommendations 1 and 3 of the Triple Impact Report and thematic area 2 of the Strategic Directions for Nursing and Midwifery. Leadership is central in the discussions about the contributions of Nursing to more effective health policies that meet the health needs of individuals and communities: leadership of the professional team, leadership in defense of gender equality with equal wages and opportunities, leadership to exercise the full potential of nursing, leadership for active participation in the processes of establishing health policies and health decision-making. Thus, the goal is the strengthening of nursing leadership, enabling more efficient and effective management and governance environments. Nursing leaders should devote time and effort to improve the social capital of the workplace in favor of their team, aiming at positive deployments of this investment^(^ 21 ^)^. If, for various reasons, investments in leadership are needed, at all levels and modalities of nursing education, ranging from undergraduate to postgraduate education, in addition to continuing education courses on specific topics, the aging of the nursing workforce and the imminent vacancies in leadership need to be considered, which need to be filled by prepared young talents.

Efforts are urgently needed to enhance the prospection of candidates for leadership posts and concern with other determining factors, such as that generation Y nurses consider or reject leadership roles^(^ 22 ^)^. Nevertheless, training and empowerment initiatives for leaders at the front line of services are needed^(^ 23 ^)^, as well as many other forms of development of leadership and the meanings of engagement, empowerment and job satisfaction^(^ 24 ^-^ 25 ^)^. Goal 4 drives nurses to appropriate the right and duty to emphasize their participation in leadership positions and to create opportunities to ensure the development of professionals and the profession at all levels.

Finally, goal 5 seeks to provide an expanded evidence base for decision makers and policy makers on how and when nursing can have the greatest impact and what prevents nurses from reaching their potential to manage these obstacles. It is directly related to Recommendations 2 and 3 of the High Level Commission on Health Employment and Economic Growth, Recommendation 5 of the Triple Impact Report and thematic area 4 of the Strategic Directions for Nursing and Midwifery. That explains the relevance of seeking evidence as to how and when the purposes of political activity are successful, ensuring greater and better participation of nursing leaderships in health policy-making, health planning and interventions, as well as in situations of frustration and failure, knowing what obstacles remain to be overcome in order to ensure this intended and necessary political activity; as the most present profession in the entire health system, 24 hours *per* day, seven days *per* week, it is responsible for pointing out feasible routes and strategies to solve problems and achieve the goals the team that outlined, representing the profession with her participation and vision. In this context, this objective synthesizes much of the other four objectives, reinforcing the need for reliable evidence to strengthen the political mobilization of Nursing, culminating in better indicators of health, economic development and gender equality.

The analysis of these interrelations reveals that the objectives of the campaign idealize a nurse profile with technical and political skills to make the difference in any work environment, with leadership and health policy-making competencies, scientific evidence of the effectiveness of its practice being disseminated to the entire society.

The limitations of this research are related to the ability of abstraction the methodology used can grant to the research problem because, being a documentary, descriptive research, other analyses of how the narrative about the topics in the data analysis process may complement and deepen possibilities to better understand the possible impact of the Nursing Now campaign, based on its connections with the factors signaled in the documents that fostered its existence and development.

Furthermore, the incipient nature of research on the theme results in limited explorations of contextual factors that may be involved in the development of the campaign goals.

## Conclusion

At this unique moment for global Nursing, this article was able to demonstrate the aspects embodied in the documents that converged to the planning, launch and development of the Nursing Now campaign, thus revealing the foundations that validate the campaign goals. Therefore, this study points to a complex process, based on evidence published by respected organizations, that the challenges for global health and its socioeconomic implications require that Nursing is acknowledged, equipped and valued to meet the current and future health demands of humanity, in different contexts.

It should be emphasized, however, that this research did not aim to analyze the implications of the terminologies international entities use concerning universal health coverage or access. Therefore, other theoretical and methodological approaches are due for this purpose.

In addition, the investments made now, advocating for changes and actions expected and explained in the Nursing Now campaign goals, will revert, of course, in deployments of the campaign with short, medium and long-term results. To achieve this success Nursing, Health Systems and the society that receives health care deserve, it is necessary that each of us, who practice nursing today, are continuously mobilized in favor of this cause and that we are all collaborators to achieve the objectives of the Nursing Now campaign.
